# Resuscitation in Threatened Death from Chloroform

**Published:** 1891-05

**Authors:** F. T. Miles

**Affiliations:** University of Maryland.


ARTICLE VII.

### RESUSCITATION IN THREATENED DEATH FROM CHLOROFORM.

PROF. F. T. MILES, UNIVERSITY OF MARYLAND.

It being now generally conceded that the best means of resuscitating patients threatened with death during the inhalation of chloroform is to invert the position of the body, it is a matter of interest to consider how this change of position accomplishes the result aimed at. The explanation usually given, that the blood is sent in increased amount to the centers innervating the heart and the apparatus of respiration, seems unsatisfactory, when we reflect that the cranial cavity is so nearly a completely closed one that the amount of its fluid contents can scarcely be expected to vary to any considerable extent through gravitation. Again, if the heart has ceased contracting, the effect of gravity will be principally on the blood in the veins, diminishing its onward flow, and thus tending to arrest the movement of the blood in the capillaries. Now we know that stopping the movement of the capillary blood is one of the most prompt and effectual means of destroying the activity of the nerve centers. Again, the danger of life from the action of chloroform arises from stopping (I do not say paralizing) the heart, and while we know of a cardio-inhibitory center in the medulla oblongata, the excitement of which will slow or stop the heart, we know of none whose excitement will increase its strength or arouse its activity. We must therefore look for some other explanation of the beneficial effects of the inverted position.

It appears to me the strikingly good effect obtained by inverting the position of the body results from the direct excitation of the heart caused by the flow of blood into the right auricle (and ventricle), which takes place when the body is in that position.

The liver is roughly computed to contain one-fourth of the blood in the whole body. The length of the vena cava inferior, from the point where the hepatic veins open into it to its ending in the right auricle, is quite inconsiderable, and it is held open by its adherence to the comparatively unyielding substance of the liver. It is further kept patulous by its attachment to the tendon of the diaphragm just before emptying into the auricle. The hepatic veins, in their course through the liver to empty into the vena cava, cannot collapse, being adherent to the hepatic substance. All this is favorable to the pouring, so to speak, of the blood from the liver into the right auricle and ventricle when the body is inverted. We know from experiment that one of the most potent means of exciting the excised frog's heart to renewed activity, after it has ceased to beat, is to fill its chambers with blood or other fluids, and I do not see why the sudden distension of the auricle in the human body should not act in a similar manner.

Moreover, it is not impossible that a mechanical influence may contribute to the beneficial effects, inasmuch as the inversion of the body is brusquely made, and thus the sudden pressure of the heavy liver against the diaphragm and heart may act as a mechanical stimulant to the latter.

I have seen one case at least in which, during the inhalation of chloroform, death seemed imminent from stopping of the heart, and in which the organ was stimulated to contraction by pretty heavy blows administered with the open palm over the cardiac regions.—*Medical Record.*

